# Anatomical Refinement of Pelvic Lymphadenectomy in Extraperitoneal Radical Prostatectomy Based on a Constant Venous Landmark

**DOI:** 10.3390/jcm15010156

**Published:** 2025-12-25

**Authors:** Bogdan Petrut, Roxana Andra Coman, Sara Arif-Miscov, Bogdan Coste, Teodor Maghiar

**Affiliations:** 1 Department of Urology, “Iuliu Hatieganu” University of Medicine and Pharmacy, 400012 Cluj-Napoca, Romania; 2 Department of Urology, Transilvania Hospital, 400486 Cluj-Napoca, Romania; 3 Department of Urology, MedLife Humanitas Hospital, 400664 Cluj-Napoca, Romania; 4 Department of Radiology, Aalborg University Hospital, 9000 Aalborg, Denmark; 5 Department of Urology, Pelican Hospital, 410450 Oradea, Romania

**Keywords:** prostate cancer, pelvic lymph node dissection, surgical landmarks, extraperitoneal radical prostatectomy, lymphocele, surgical morbidity

## Abstract

**Background/Objectives**: Extended pelvic lymph node dissection (ePLND) is the standard approach for staging intermediate- and high-risk prostate cancer (PCa), but the optimal extent of the procedure is still being debated due to the need to balance staging benefits with postoperative complications. This study aimed to assess whether the deep circumflex iliac vein, a consistent anatomical venous landmark, can guide a more selective dissection template that maintains staging accuracy while reducing morbidity. **Methods**: We conducted a retrospective analysis of 32 patients with intermediate- or high-risk PCa and histologically confirmed nodal metastases who underwent minimally invasive extraperitoneal radical prostatectomy with ePLND between 2018 and 2024. The lymph nodes located above (supra-venous) and below (infra-venous) the landmark vein were dissected separately and analysed histologically. Postoperative lymphoceles and related complications were recorded. **Results**: No metastatic lymph nodes were found in the subvenous region across all patients. All positive nodes were located cranially to the landmark, primarily in the obturator, internal iliac and proximal external iliac regions. Lymphoceles occurred in all patients, 62.5% of whom were symptomatic, 43.8% of whom had a fever, and 18.8% of whom were septic and required drainage. Four patients underwent laparoscopic reintervention for recurrent lymphoceles. **Conclusions**: The absence of metastatic involvement in the subvenous region suggests it is an oncologically low-yield zone. A refined dissection template omitting this area, guided by a reproducible venous landmark, may lower complication rates without compromising staging accuracy. Prospective validation is warranted before clinical adoption.

## 1. Introduction

The standard approach for the surgical treatment of intermediate- and high-risk prostate cancer (PCa) is radical prostatectomy (RP) with extended pelvic lymph node dissection (ePLND). This procedure offers prognostic and therapeutic benefits, including accurate staging and the removal of potential micrometastatic disease. According to the most recent guidelines from both the European Association of Urology and the National Comprehensive Cancer Network, ePLND is recommended for patients with an estimated risk of lymph node involvement exceeding predefined thresholds as calculated using validated predictive nomograms [1,2]. However, the oncologic benefits of more extensive dissection must be weighed carefully against the increased risk of perioperative morbidity [3]. Despite its established role in staging, the optimal extent of ePLND and its associated morbidity profile are subjects of ongoing refinement [4,5]. While diagnostic accuracy improves with broader dissection, this comes at the cost of significantly increased perioperative morbidity. Lymphoceles are one of the most frequent and clinically significant complications [4]. Studies report imaging-detected rates of 30–60% and symptomatic rates of 5–20%, particularly when prophylactic drainage is absent [6,7]. Symptomatic lymphoceles may result in infection, pelvic pain, compression of vascular structures, and delayed recovery [5,8]. Therefore, strategies that optimize the extent of lymph node dissection while maximizing oncologic efficacy and minimizing surgical morbidity are clinically relevant. The extent of dissection, particularly into anatomically susceptible areas with a high concentration of lymphatics, is increasingly recognised as a significant modifiable risk factor for these complications [9]. Therefore, a key objective of optimising surgical management is to determine the minimum anatomical template required for accurate staging, while minimising dissection in zones with low yield and high morbidity [10,11].

The extraperitoneal approach to RP is increasingly utilized due to its lower risk of operative complications [12]. Importantly, this approach involves distinct anatomical and lymphatic considerations compared to the transperitoneal route. However, there is limited evidence regarding the precise anatomical boundaries that must be included in an ePLND to ensure adequate staging without overextending dissection into low-yield areas [13].

However, the distinct anatomical landscape of the extraperitoneal pelvis means that dissection boundaries must be considered carefully within this approach. Based on our observations during surgery, we identified the deep circumflex iliac vein (DCIV), a consistent tributary of the external iliac vein that runs through the lower part of the iliac fossa, as a reproducible landmark that can be used to mark this low-yield zone during extraperitoneal dissection. Metastatic involvement of lymph nodes located distal to the DCIV appears exceptionally uncommon in patients with intermediate- and high-risk PCa undergoing primary surgery.

This prospective observational (non-interventional) study aimed to evaluate the distribution of histologically confirmed lymph node metastases in a cohort of node-positive patients who underwent extraperitoneal RP with ePLND, specifically assessing the yield of nodes located caudal to the DCIV. A secondary aim was to investigate the potential association between dissection extending below the DCIV and the incidence of postoperative lymphocele formation and related complications. We hypothesized that the DCIV reliably demarcates the distal boundary of oncologically relevant lymphatic tissue in this setting and that omitting dissection below this landmark could significantly reduce morbidity without compromising staging accuracy.

## 2. Materials and Methods

This prospective observational (non-interventional) single-center study included 32 patients who were diagnosed with intermediate- or high-risk PCa according to the D’Amico classification [14] and had positive lymph nodes after surgery. Between January 2018 and December 2024, these patients underwent extraperitoneal RP with ePLND. An experienced surgeon performed all procedures using either the da Vinci X or Xi Robotic Surgical System (Intuitive Surgical, Sunnyvale, CA, USA) or a 3D laparoscopic approach.

The inclusion criteria consisted of histologically confirmed prostate adenocarcinoma with at least one positive lymph node according to the final pathology report. All patients underwent anatomically defined ePLND. In each case, the DCIV, which was present in the majority of patients, was used as an anatomical landmark to separate lymph node packets intraoperatively.

**Figure 1:**
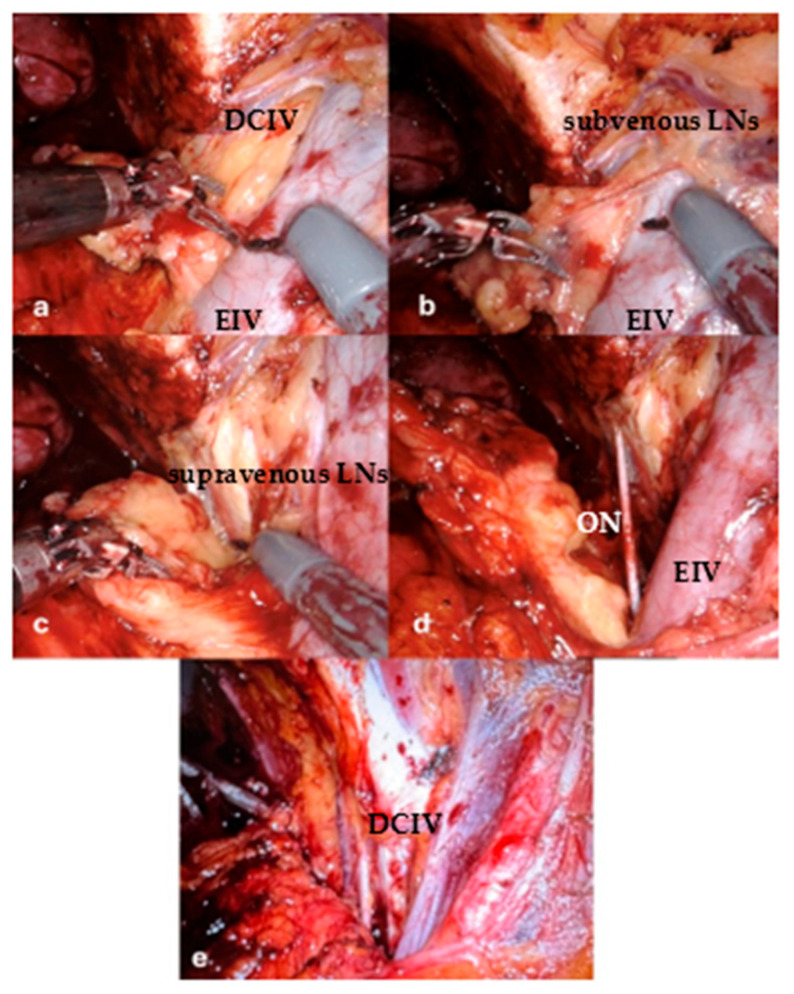
DCIV—deep circumflex iliac vein, EIV—external iliac vein, subvenous LNs—subvenous lymph nodes, supravenous LNs—supravenous lymph nodes, ON—obturator nerve. (**a**) The DCIV (double in this case) that divides the lymph nodes in supra and subvenous groups, (**b**) Subvenous group is excised, and the dissection continues with supravenous lymph nodes, (**c**) Excising the obturatory lymph nodes, part of the supravenous group, (**d**) Supravenous lymph nodes are also excised; the obturator fossa is exposed, (**e**) The final aspect of the LND.

The lymphatic tissue located below the venous landmark (the “subvenous group”) was marked with a metal clip during the operation and submitted separately for histopathological analysis. The rest of the dissection (“supravenous group”) followed the standard anatomical boundaries of ePLND (Figure 1). This delineation was performed to evaluate whether the subvenous area harbored any metastatic lymph nodes and whether omitting this area from the dissection could reduce the rate of lymphatic complications.

Postoperative outcomes, including the incidence of lymphoceles, infections and inflammatory complications, were assessed through clinical evaluation and routine imaging (ultrasound or CT), performed within 3 weeks of surgery. Although not formally quantified, subjective intraoperative and postoperative observations regarding lymphocele size were also recorded.

### Statistical Analyses

Statistical analyses were conducted to evaluate the relationship between anatomical lymph node dissection zones, nodal positivity, and histopathological tumor grade.

A Wilcoxon signed-rank test was used to compare lymph node yield between the supravenous and subvenous anatomical regions. A binomial exact test was performed to determine if the absence of metastatic involvement in the subvenous region was due to chance, thus evaluating the oncologic relevance of this anatomical zone. To explore the correlation between tumor aggressiveness and nodal metastatic burden, Spearman’s rank correlation analysis was used to test the association between Gleason score and the number of positive lymph nodes.

All statistical tests were two-sided, and a *p*-value of less than 0.05 was considered statistically significant. Analyses were conducted using IBM SPSS Statistics, version 29.0 (IBM Corp., Armonk, NY, USA) and R software, version 4.3.1 (R Foundation for Statistical Computing, Vienna, Austria).

## 3. Results

### 3.1. Patient Characteristics

Between January 2018 and December 2024, a total of 32 patients with intermediate- or high-risk PCa underwent extraperitoneal RP with ePLND. 18 patients were treated using a robot-assisted approach, and 14 underwent 3D laparoscopic surgery. All patients had at least one positive lymph node confirmed by histology.

The patients’ ages ranged from 52 to 73 years, with a median of 63 (IQR: 60–67) years. The patients had a mean BMI of 28.6 ± 2.2 kg/m^2^ (range: 23.3–32.7). Patients’ demographic characteristics are illustrated in Table 1. The median preoperative PSA was 13.8 ng/mL (IQR: 8.0–17.9 ng/mL). The Gleason score distribution is presented in Table 2.

### 3.2. Descriptive Lymph Node Data

The number of lymph nodes removed varied by anatomical region and oncological burden, as detailed in Table 3. No metastatic lymph nodes were identified in the subvenous region in any patient.

### 3.3. Statistical Analysis

Three statistical analyses were conducted to evaluate the relationship between anatomical dissection zones, nodal yield, metastatic involvement, and tumor aggressiveness.

A Wilcoxon signed-rank test comparing lymph node counts in the supra- and subvenous regions revealed a statistically significant difference (*p* < 0.0000000005). On average, 13.3 ± 3.81 lymph nodes were retrieved from the supravenous zone (range: 7–21), whereas only 2.4 ± 0.95 nodes (range: 1–4) were removed from the subvenous zone. This indicates that the supravenous region consistently yielded a significantly higher nodal count.

A binomial test was performed to evaluate the absence of positive lymph nodes in the subvenous region among all patients. The test yielded a highly significant result (*p* < 0.0000000003), which strongly suggests that this anatomical region is a low-yield zone for metastatic involvement in patients with intermediate- and high-risk PCa.

**Figure 2:**
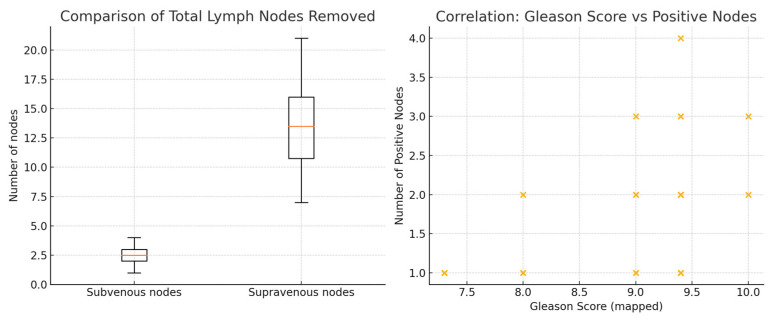
Spearman’s rank correlation—Gleason Score vs. Positive Nodes.

Lastly, Spearman’s rank correlation analysis revealed a positive correlation between Gleason score and the number of positive lymph nodes (*p* = 0.36), reaching statistical significance (*p* = 0.045) is represented in Figure 2. These results suggest that patients with higher-grade tumors tend to have a greater burden of nodal metastasis.

### 3.4. Anatomical Distribution of Positive Lymph Nodes

In all 32 cases, ePLND was performed both cranially and caudally to DCIV, which was identified intraoperatively and used as an anatomical landmark. The lymphatic tissue located below this landmark—the “subvenous group”—was marked with a metal clip and sent for separate histopathological analysis.

Histological evaluation revealed that none of the 32 patients had positive lymph nodes in the subvenous region. All metastatic nodes were located in areas cranial to the venous landmark—namely the obturator fossa, internal iliac, and external iliac territories. This consistent absence of nodal involvement below the reference point suggests that the subvenous territory may represent an oncologically low-yield region in intermediate- and high-risk PCa when approached extraperitoneally.

### 3.5. Lymphocele Formation and Postoperative Complications

All patients underwent pelvic ultrasound between postoperative days 8 and 21, coinciding with Foley catheter removal. Lymphocele formation was identified in 100% of patients (32/32), with fluid collections predominantly located adjacent to the external iliac vessels or within the paravesical space. The clinical outcomes associated with lymphocele formation are summarized in Table 4.

• 12 patients (37.5%) had asymptomatic lymphoceles that required no further intervention.
• 20 patients (62.5%) experienced pelvic pain consistent with symptomatic lymphoceles.
  ○ Among these, 14 patients (43.8%) developed febrile episodes, which were managed successfully with conservative treatment, including antibiotics and anti-inflammatory agents.
  ○ 6 patients (18.8%) progressed to sepsis unresponsive to conservative therapy, necessitating surgical percutaneous drainage.
▪ Of these, 4 patients experienced recurrent lymphoceles accompanied by renewed inflammatory or septic symptoms. These patients subsequently underwent laparoscopic drainage with peritonealization of the lymphocele cavity to prevent recurrence.

## 4. Discussion

ePLND remains the gold standard for accurately staging nodal disease in patients with intermediate- and high-risk PCa, as recommended in major guidelines [1,2]. While its therapeutic benefit is debated, its diagnostic superiority over limited dissection is undisputed, as it significantly reduces understaging and informs adjuvant therapy decisions [11]. However, ePLND carries substantial morbidity, including increased operative time and blood loss. Notably, it carries a high risk of lymphoceles (reported at 5–61%) and related complications [8,15].

In this study, all patients underwent extraperitoneal ePLND using a 3D laparoscopic or robotic-assisted approach. We systematically identified an anatomical landmark—DCIV. The lymph nodes caudal to this landmark (‘subvenous group’) associated with lower extremity drainage were dissected and analysed separately. Significantly, no positive lymph nodes were found within this subgroup (0/32 patients), despite all patients having confirmed nodal metastases elsewhere. This finding is consistent with the current understanding of PCa lymphatic spread. For example, Fossati et al. emphasized that, although extended dissection improves staging accuracy, its therapeutic benefit may plateau beyond the obturator and internal iliac regions [5]. Similarly, Dong et al. questioned the potential oncological benefits of pelvic lymph node dissection for PCa, recommending identifying the most suitable excision range and emphasising the importance of predictive models [16].

Mapping studies confirm that metastatic involvement predominantly occurs within the obturator fossa, the internal iliac region and the proximal external iliac region. The distal external iliac nodes represent a low-yield zone that is rarely involved, except in cases of massive nodal burden [17].

In our cohort, lymphoceles were evident in all patients (100%) on postoperative imaging performed between days 8 and 21. Of these, 62.5% were painful and symptomatic, and 43.8% developed feverish episodes that were managed conservatively. Notably, six patients (18.8%) developed sepsis requiring percutaneous drainage, four of whom required laparoscopic drainage with peritonealization due to recurrence. These rates are higher than those reported in many large studies [4,7,8], suggesting that uniform dissection of the subvenous region with its rich and delicate lymphovascular structures may have contributed significantly to this morbidity.

Statistical analysis reinforced the anatomical and clinical relevance of our findings. The Wilcoxon signed-rank test showed a highly significant difference between supravenous and subvenous nodal yields, with markedly more lymph nodes retrieved above the vein—consistent with established pelvic lymphatic drainage pathways. A binomial test confirmed that no metastatic nodes were found in the subvenous region in any of the 32 patients, making a chance distribution extremely unlikely and indicating that this area provides no meaningful staging information in intermediate- and high-risk PCa treated extraperitoneally. Finally, the significant positive correlation between Gleason score and the number of metastatic nodes aligns with the known association between tumour grade and metastatic burden. Notably, even in patients with Gleason ≥ 9 disease, no metastases were detected below the venous landmark, suggesting that increased tumour aggressiveness does not extend nodal spread into the subvenous territory within this template.

Overall, these results strengthen the rationale for anatomically refined lymphadenectomy strategies that prioritise oncological efficacy while aiming to minimise procedural morbidity.

Although no definitive conclusions can be drawn about the origin of each lymphocele due to the uniformity of the dissection template, the consistent absence of metastatic involvement in the subvenous group in a high-risk population raises an important question: can this area safely be omitted to reduce morbidity without compromising oncological outcomes? Based on our findings, we propose a modified ePLND template, which omits dissection caudal to the DCIV in selected patients. This anatomical refinement may optimise the balance between staging accuracy and complication prevention.

LND is a critical component of managing patients with PCa. Understanding the indications, drainage patterns, extent, and total volume of LND, as well as the utilization of nomograms and genomics, and the complication profile, can aid in the creation of an individualized template. The combination of newer imaging modalities with molecular-guided robotic surgery has personalised the approach to LND, achieving excellent safety, efficacy and oncological outcomes for these patients [18]. Furthermore, novel imaging technologies such as PSMA PET/CT have been shown to have unparalleled sensitivity and specificity when it comes to identifying nodal metastases, even in cases of small-volume disease [19]. Complementary imaging modalities may further refine nodal mapping. A recent retrospective study comparing PSMA PET/CT with ferumoxtran-10 nanoparticle–enhanced MRI (nano-MRI) showed that nano-MRI detected significantly more suspicious lymph nodes, particularly smaller ones [20]. Integrating functional imaging into surgical planning could allow for the creation of personalised lymphadenectomy templates that are anatomically and biologically tailored [21]. Furthermore, surgical innovations such as real-time fluorescent lymphatic mapping and robot-assisted sentinel node dissection offer the prospect of even more selective interventions in the future [22,23].

This study has several important limitations. Firstly, single-centre design and reliance on the experience of a single surgeon may restrict the generalisability of the findings. The modest sample size further constrains the statistical power and robustness of the conclusions.

Secondly, the absence of long-term oncological follow-up means that it is not possible to make any definitive assessments regarding the impact of omitting the subvenous nodal region on key outcomes such as biochemical recurrence or cancer-specific survival. Thirdly, while the DCIV was a reliable intraoperative landmark in this cohort, its anatomical consistency across broader patient populations has yet to be validated. Prospective, multi-institutional studies with longer follow-up periods are essential to confirm the oncological non-inferiority of this modified dissection template, and to establish whether it can reduce postoperative morbidity without compromising staging accuracy.

## 5. Conclusions

This study identifies the deep circumflex iliac vein as a reliable anatomical landmark that defines a pelvic nodal region with minimal metastatic involvement in cases of intermediate- and high-risk prostate cancer. Dissection below this vein was found to increase morbidity, particularly the rates of lymphocele formation, infection, sepsis and re-operation, without improving staging accuracy. Therefore, avoiding subvenous dissection during extraperitoneal radical prostatectomy may reduce complications while preserving oncological validity. Incorporating this landmark provides a reproducible, anatomy-based refinement of the extended pelvic lymph node dissection template. However, larger prospective studies, randomised trials and imaging-guided nodal mapping (PSMA PET/CT), as well as intraoperative navigation using fluorescent imaging, are required to confirm its safety, long-term oncological impact and applicability in routine practice.

## Figures and Tables

**Table 1:** Patients’ demographic characteristics.

Patients’ Characteristics	
Age	63 (IQR: 60–67) years
Comorbidities	
Hypertension	84.37%
Diabeus	34.37%
Medical use	
Alfa-blocants	87.5%
5-ARI	21.87%
BMI	28.6 ± 2.2 kg/m^2^ (range: 23.3–32.7)
Family history—first degree relative	28.12%

**Table 2:** Gleason score distribution.

Gleason Score	No Patients
10 (5 + 5)	2
9 (5 + 4)	19
9 (4 + 5)	5
8 (4 + 4)	3
7 (4 + 3)	3

**Table 3:** Lymph node yield and metastatic involvement.

Lymph Node Region	Mean ± SD	Median (Range)
Subvenous Nodes	2.4 ± 0.95	2.5 (1–4)
Supravenous Nodes	13.3 ± 3.81	13.5 (7–21)
Positive Lymph Nodes	1.72 ± 0.81	2.0 (1–4)

**Table 4:** Clinical outcomes of lymphocele formation (*n* = 32).

Clinical Outcome	No of Patients	Percentage (%)
Lymphocele detected on ultrasound	32	100.0
Asymptomatic lymphocele (no intervention required)	12	37.5
Symptomatic lymphocele (pelvic pain)	20	62.5
With febrile episodes (conservatively managed)	14	43.8
Progressed to sepsis (required percutaneous drainage)	6	18.8
Recurrence post-drainage (required laparoscopy)	4	12.5

## Data Availability

Anonymized data may be available from the corresponding author upon reasonable request.

## Abbreviations

The following abbreviations are used in this manuscript:

PCa	Prostate Cancer
RP	Radical Prostatectomy
ePLND	extended Pelvic Lymph Node Dissection
DCIV	Deep Circumflex Iliac Vein
